# Sex-Related Neuroanatomical Basis of Emotion Regulation Ability

**DOI:** 10.1371/journal.pone.0097071

**Published:** 2014-05-16

**Authors:** Feng Kong, Zonglei Zhen, Jingguang Li, Lijie Huang, Xu Wang, Yiying Song, Jia Liu

**Affiliations:** 1 State Key Laboratory of Cognitive Neuroscience and Learning & IDG/McGovern Institute for Brain Research, Beijing Normal University, Beijing, China; 2 School of Psychology, Beijing Normal University, Beijing, China; 3 Center for Collaboration and Innovation in Brain and Learning Sciences, Beijing Normal University, Beijing, China; George Mason University/Krasnow Institute for Advanced Study, United States of America

## Abstract

Behavioral research has demonstrated that males have a higher capability of regulating their own and others' emotions than females; however, little is known about the sex-specific brain mechanisms involved in emotion regulation ability. In the present study, we used voxel-based morphometry to investigate the neural basis underlying emotion regulation ability in a large sample of young adults. Assessment of emotion regulation ability was performed using the Wong and Law Emotional Intelligence Scale. As expected, males significantly scored higher in emotion regulation ability than females did. More importantly, we found the sex differences in the neuroanatomical basis of emotion regulation ability. Males showed a stronger positive relation between emotion regulation ability and regional gray matter volume (rGMV) in the right dorsolateral prefrontal cortex. In contrast, females demonstrated a stronger positive relation between emotion regulation ability and rGMV in an anatomical cluster that extends from the left brainstem to the left hippocampus, the left amygdala and the insular cortex. The present study provides the first empirical evidence regarding the sex-linked neuroanatomical correlates of emotion regulation ability. These findings may help understand why there is a higher prevalence of affective disorders in females and maladaptive behaviors in males.

## Introduction

According to John Gray's best-seller, Men Are from Mars, Women Are from Venus, males and females not only communicate differently but also think, feel, perceive, respond, love, and appreciate differently [1]. Such differences can be reflected in the domain of emotion regulation. Emotion regulation refers to a person's ability to regulate one's own and others' emotional states and is regarded as a crucial component of emotional intelligence [2]. Numerous behavioral studies have suggested that males score higher than females with regard to self-report measurement of emotion regulation ability and other similar constructs. For example, using the Trait-Meta Mood Scale (TMMS), Amelang and Steinmayr (2006) and Extremera, Durán and Rey (2007) found that males had higher mood repair scores [3], [4]. Using the Wong and Law Emotional Intelligence Scale (WLEIS), Kong, Zhao and You (2012) found that males scored higher in emotion regulation ability [5]. Using the Trait Emotional Intelligence Questionnaire (TEIQue), Mikolajczak, Luminet, Leroy and Roy (2007) found that males had higher self-control scores [6], and finally, using the Emotional Quotient Inventory (EQI), Bar-On, Brown, Kirkcaldy and Thomé (2000) found that males scored higher in stress tolerance and impulse control [7]. However, some studies found inconsistent results in the sex differences in emotional intelligence [6], [8], [9], [10]. For example, using Brain Resource Inventory for Emotional Intelligence Factors (BRIEF), Craig et al. (2009) found that females scored higher on Empathy than males, whereas males scored higher on Self-concept, so females scored higher on the total scale than males. Using the Trait Emotional Intelligence Questionnaire, Mikolajczak et al. (2007) found that females scored higher on Emotionality, whereas males scored higher on Self-Control and Sociability, as a result, males scored higher on the total scale than females [6]. The discrepancy may be due to the choice of measurement instrument, but these inconsistent findings are not accidental, which may reflect the gender differences in many emotional aspects. These sex differences may be explained by the “extreme male brain theory of autism” proposed by Baron-Cohen (2002). According to this theory, the masculine brain predominantly seeks to understand and construct systems (i.e., “systemize”), whereas the feminine brain is predominantly structured to feel empathy (i.e., “empathize”) [11]. Here we focused on emotional regulation ability and used magnetic resonance imaging (MRI) to investigate the sex-specific neural basis of this ability.

Previous functional neuroimaging studies have mainly focused on emotion regulation strategies (e.g., cognitive reappraisal); such studies have demonstrated that both subcortical regions and cortical regions are involved in emotion regulation strategies. Subcortical regions include the amygdala, hippocampus and cerebellum [12], [13], [14], [15], [16], [17]. Cortical regions include the prefrontal cortex (dorsolateral prefrontal cortex (DLPFC), ventromedial prefrontal cortex (VMPFC), orbitofrontal cortex (OFC), and the anterior cingulate cortex (ACC)) and the insular cortex [12], [13], [15], [18], [19]. However, emotion regulation ability is differentiated from emotion regulation strategies because emotion regulation ability reflects effective choices and flexible application of different strategies for managing emotionally charged situations. Recently, several structural neuroimaging studies have explored the neuroanatomical basis of emotion regulation ability, but the results are less clear. For instance, Killgore et al. (2012) found that emotion regulation ability correlated positively with regional gray matter volume (rGMV) in the bilateral VMPFC [20], while Takeuchi et al. (2011) found that emotion regulation ability correlated with regional gray matter density (rGMD) in the right anterior insula, the right cerebellum, the precuneus, and the medial prefrontal cortex [21]. Koven, Roth, Garlinghouse, Flashman and Saykin (2011) found that better mood repair was related to larger rGMV in three clusters in frontal and inferior parietal areas [22]. Despite the limited statistical power caused by the relatively small sample size used in these studies, we speculate that sex differences may be another significant factor in these inconsistent findings.

To our knowledge, there are three functional neuroimaging studies that have explored sex-related differences on cognitive reappraisal strategies ([23], [24], [25], for a review see Ref. [26]). Using an effortful reappraisal task, in which participants consciously maintained or down-regulated their negative emotions, two of the studies reported stronger prefrontal activity in males [23], [25], while the third study found stronger prefrontal activity in females [24]. In addition, the third study reported that the amygdala was more activated in females [24], while the two other studies failed to find an effect of gender on amygdala activity [23], [25]. One possible reason for this inconsistency is that the regulation instructions for participants varied across the studies, and thus different types and number of reappraisal strategies may have been used. Although the results are mixed, these findings indicate distinct roles for prefrontal and subcortical regions in emotion regulation between males and females. However, to the best of our knowledge, no study has directly explored the sex-linked neuroanatomical correlates of emotion regulation ability.

To investigate this issue, here we extended the previous studies in three ways. First, we assessed each participant's general ability to regulate emotions in daily life using a well-established self-report questionnaire (WLEIS) [27]. Second, we used voxel-based morphometry (VBM) to examine structural differences underlying emotion regulation ability in a large sample size of males and females (N = 299; 159 females). Functional and structural MRI studies are believed to complement each other, but structural MRI studies are particularly suitable for describing the neural correlates of emotion regulation ability because the short reappraisal tasks in functional MRI studies may hardly tap the cognitive-affective processes involving the whole range of emotion regulation ability. Finally, a large sample size of young adults was used in this study, which will provide a higher statistical power to test and identify the sex-specific neural correlates of emotion regulation ability. Given that males have better emotion regulation skills than females, we hypothesized that males and females would show differences with regard to the brain regions involved in emotion regulation ability. Specifically, we hypothesized that males' emotion regulation ability would be more strongly associated with rGMV in cortical regions, particularly in the prefrontal cortex, which is associated with cognitive processes [28], [29]. Similarly, females would mainly have a stronger association between emotion regulation ability and rGMV in regions (e.g., the amygdala) that are implicated in emotional processes [30], [31].

## Methods

### Participants

College students (N = 299; 159 females; mean age  = 21.55 years, SD  = 1.01) from Beijing Normal University, Beijing, China, participated in this study. Both behavioral and MRI protocols were approved by the Institutional Review Board of Beijing Normal University. Written informed consent was obtained from all participants prior to the experiment. Two participants were excluded due to missing items or erroneous reports in the questionnaire. Another five participants were removed from further analyses due to extraordinary scanner artifacts or abnormal brain structures (e.g., unusually large ventricles). Thus, 292 participants contributed to our study findings (133 males, mean age  = 21.57 years, SD  = 1.00; 159 females. mean age  = 21.54 years, SD  = 1.02).

### Psychological measurement

Participants' emotion regulation ability was assessed using the 4-item Regulation of Emotion (ROE) scale of the WLEIS [26]. The ROE scale measures individuals' ability to regulate their emotions and their ability to quickly recover from psychological stress; sample items include, “I am quite capable of controlling my own emotions” and “I can always calm down quickly when I am very angry.” Participants were then instructed to indicate the extent to which they agree or disagree with each statement using a 7-point Likert-type scale. Higher scores in the ROE scale indicate better emotion regulation ability. The scale has been demonstrated to have high internal consistency, convergent/discriminant validity with related constructs of loneliness, positive affect, negative affect, depression, and empathy, and good concurrent validity with other emotion regulation measures, including the Optimism/Mood Regulation subscale of the Schutte' Emotional Intelligence Scale, the Repair of Emotion subscale of Trait Meta-Mood Scale, and the Emotional Control Questionnaire [32], [33], [34].

### MRI acquisition

Participants were scanned using a Siemens 3T scanner (MAGENTOM Trio, a Tim system) with a 12-channel phased-array head coil at BNU Imaging Center for Brain Research, Beijing, China. MRI structural images were acquired using a 3D magnetization prepared rapid gradient echo (MP-RAGE) T1-weighted sequence (TR/TE/TI  = 2530/3.39/1100 ms, flip angle  = 7 degrees, FOV  = 256×256 mm). One hundred and twenty-eight contiguous sagittal slices were acquired with 1×1-mm in-plane resolution and 1.33-mm slab thickness for whole brain coverage.

### Image Processing for VBM

VBM was employed to characterize the neuroanatomical differences in gray matter volume (GMV) and the neuroanatomical correlates of behavioral performance across participants [35]. In this study, VBM was performed using SPM8 (Statistical Parametric Mapping, Wellcome Department of Imaging Neuroscience, London, UK), with an optimized VBM protocol [36] on T1-weighted structural MRI images. First, image quality was assessed by manual visual inspection. Five participants whose images had excessive scanner artifacts or showed gross anatomical abnormalities were excluded. Second, the origin of the brain was manually set to the anterior commissure for each participant. Third, images were segmented into four distinct tissue classes: gray matter, white matter, cerebrospinal fluid, and everything else (e.g., skull and scalp) using a unified segmentation approach [37]. Forth, the MNI152 template was used to spatially normalize the gray matter images for each participant using the Diffeomorphic Anatomical Registration through Exponential Lie algebra (DARTEL) registration method [38]. DARTEL registration involves repetitively computing the study-specific template based on the average tissue probability maps of all participants and then warping all participants' tissue maps into a generated template to improve the alignment. Fifth, gray matter voxel values were modulated by multiplying the Jacobian determinants derived from the normalization procedure to preserve the volume of tissue from each structure after warping. The modulated gray matter images were then smoothed using an 8-mm full width at half maximum (FWHM) isotropic Gaussian kernel. Finally, to exclude noisy voxels, the modulated images were masked using absolute masking with a threshold of 0.2. The masked-modulated gray matter images were used for further statistical analyses.

### Statistical Analysis of VBM

Sex difference in the correlation between emotion regulation ability and rGMV was tested using the condition by covariate interaction analysis [39]. The interaction analysis treated sex as a condition, the score of emotion regulation ability as a covariate of interest, and the total GMV and age as covariates of no interest. Statistical analysis was performed using a general linear model (GLM). Multiple comparison correction was performed by setting the voxel-wise intensity threshold at *p*<0.05 and a cluster-level threshold determined by Monte Carlo simulations (10,000 iterations) conducted in the AlphaSim program within AFNI [40]. Accordingly, significant effects were reported when the volume of a cluster was greater than the Monte Carlo simulation determined minimum cluster size on whole brain GMV (i.e., 943 voxels), above which the probability of type I error was below 0.01.

To test the specificity of correlation, we also detected the neuroanatomical correlates of individual differences in emotion regulation ability in the whole sample. Statistical analysis treated total GMV, age and sex as confounding covariates and the score of emotion regulation ability as a covariate of interest. The threshold for statistical significance was also set at MC-cluster-corrected *p*<0.05 and significant effects were reported when the volume of a cluster was greater than 943 voxels.

## Results

The summed score for the ROE scale was used as an index of emotion regulation ability, whereby higher scores indicated a better ability. Demographic characteristics for the participants are presented in Table 1. As indicated in Table 1, measures of emotion regulation ability, use of emotion ability, self-emotion appraisal ability, and others-emotion appraisal ability had good internal consistency. Moreover, emotion regulation ability was moderately and positively related to use of emotion ability (*r* = 0.36, *p*<0.001), self-emotion appraisal ability (*r* = 0.44, *p*<0.001), and others-emotion appraisal ability (*r* = 0.28, *p*<0.001), thus indicating that the ROE scale has good discriminant validity. The independent sample t-test analyses revealed no significant sex differences in age (*t* = 0.29; *p* = 0.77) and use of emotion ability (*t* = 1.15; *p* = 0.25), self-emotion appraisal ability (*t* = 0.83; *p* = 0.41), and others-emotion appraisal ability (*t* =  −1.28; *p* = 0.20). Importantly, we found a significant difference in emotion regulation ability between male and female groups (*t*(290)  = 2.92, *p* = 0.004, Cohen's d = 0.34), which is consistent with previous findings that males have higher levels of emotion regulation ability than females [5], [6], [7]. Next, we examined whether the sex-specific differences in emotion regulation, as observed in the behavioral measurements, had distinct neural substrates.

**Table 1:** Demographic characteristics for participants.

Variable	Group			Sex difference		
	All	Males	Females			
Age						
Mean (SD)	21.6 (1.02)	21.6 (1.00)	21.6 (1.03)		n.s.	
Range	18–25	18–25	19–24			
Emotion regulation ability (α = 0.86)						
Mean (SD)	19.2 (4.3)	20.0 (3.8)	18.6 (4.6)		<.01	
Use of emotion ability (α = 0.69)						
Mean (SD)	21.3 (3.3)	21.5 (3.2)	21.1 (3.3)		n.s.	
Self-emotion appraisal ability (α = 0.78)						
Mean (SD)	21.9 (3.0)	22.1 (2.9)	21.8 (3.1)		n.s.	
Others-emotion appraisal ability (α = 0.91)						
Mean (SD)	20.8 (4.2)	20.4 (4.2)	21.04 (4.1)		n.s.	
DLPFC						
	0.34 (0.06)	0.37 (0.06)	0.33 (0.05)		<.001	
Hippocampus/amygadala/insular						
	0.27 (0.03)	0.28 (0.02)	0.23 (0.02)		<.001	

*Note*: n.s., not statistically significant at *p*<0.05.

**Figure 1:**
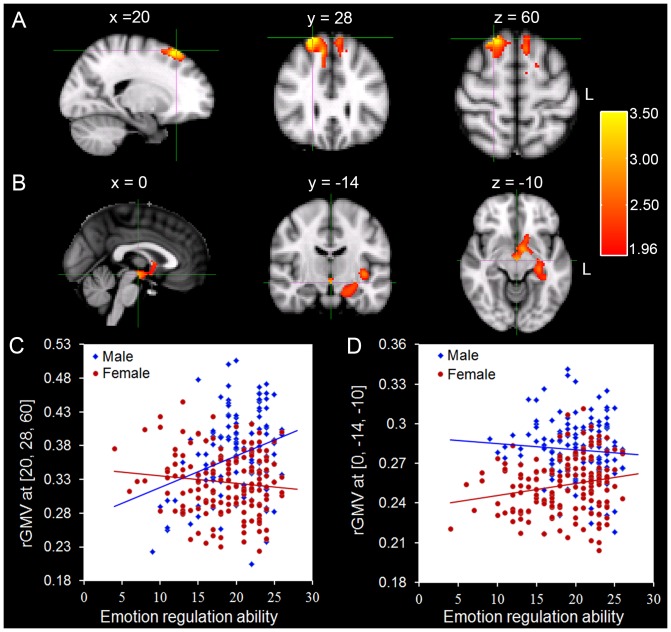
Sex-specific correlation between emotion regulation ability and regional gray matter volume. A: The right DLPFC, where the interaction between sex and emotion regulation ability was found, is rendered in the e Montreal Neurological Institute (MNI) space. B: The anatomical cluster that extends from the left brainstem to the left hippocampus, the left amygdala and the insular cortex, where the interaction between sex and emotion regulation ability was found, is rendered in the MNI space. C: Scatter plots depicting correlations between rGMV of the right DLPFC [20], [28], [60] and individual variability in emotion regulation ability in males (N = 133, r = 0.30, p<0.001) and females (N = 159, r =  −0.08, p>0.05). D: Scatter plots depicting correlations between rGMV of the anatomical cluster that extends from the left brainstem to the left hippocampus, the left amygdala and the insular cortex [0, −14, −10] and individual variability in emotion regulation ability in males (N = 133, r = 0.20, p<0.05) and females (N = 159, r =  −0.11, p>0.05).

To do this, we conducted a condition by covariate interaction analysis with sex as a condition and the score of emotion regulation ability as a covariate of interest. We revealed a significant interaction of sex by emotion regulation ability in two anatomical clusters: one in the right DLPFC (MNI coordinate: 20, 28, 60; Cluster-corrected *p*<0.01) (Fig. 1A) and the other that extends from the left brainstem to the left hippocampus, the left amygdala and the insular cortex (MNI coordinate: 0, −14, −10; Cluster-corrected *p*<0.01) (Fig. 1B). Brain structures involved in the second cluster are known as central parts of the limbic system [41]. Specifically, males showed a stronger positive relation between emotion regulation ability and rGMV in the right DLPFC (r = 0.30, p<0.001), relative to females (r =  −0.08, p>0.05) (Fig. 1C). In contrast, females showed a stronger positive relation between emotion regulation ability and rGMV in the other anatomical cluster (r = 0.20, p<0.05), relative to females (r =  −0.11, p>0.05) (Fig. 1D). The anatomical location of these clusters identified by the VBM analysis in this study was close to regions identified by fMRI using emotion regulation tasks [12], [23], [42], [43], [44]. In addition, we tested the gender differences of rGMV in these two clusters. We found that males had larger rGMV in these two clusters than females (*ps*<0.001).

To test the specificity of these correlations, we also detected the neuroanatomical correlates of individual differences in emotion regulation ability in the whole sample. A significant positive correlation between emotion regulation ability and rGMV was found in a cluster that included the left precuneus (MNI coordinate: −2, −72, 32; Cluster-corrected *p*<0.01) (Table 2). Taken together, these results suggest that the left hippocampus and the left amygdala, as well as the right DLPFC, have a sex-specific correlation with emotion regulation ability.

**Table 2:** Regions correlating with emotion regulation ability.

Region	Side	MNI coordinate			*Z*	Cluster size (mm^3^)
		*x*	*y*	*z*		
*Male > female*						
Dorsal lateral prefrontal cortex	Right	20	28	60	3.80	1658*
*Female> male*						
Hippocampus, Amygdala, insular cortex	Left					
*In total sample*						
Precuneus	Left	−2	−72	32	3.45	1753*

*Note*: MNI  =  Montreal Neurological Institute; * MC-cluster-corrected *p*<0.01.

## Discussion

The aim of the present study was to investigate the sex-linked neuroanatomical basis of emotion regulation ability in a large sample of young healthy adults. As expected, males reported higher levels of emotion regulation ability relative to females, which is consistent with previous behavioral findings that males have a greater ability to regulate emotions than females [5], [6], [7]. More importantly, VBM analysis revealed males relying more on the right DLPFC and females relying more on limbic regions including the left hippocampus, the left amygdala and insular cortex. Thus, the present study provides the first empirical evidence for sex-related neuroanatomical basis of emotion regulation ability.

Males demonstrated a stronger positive relation between emotion regulation ability and rGMV in the right DLPFC, which is in line with previous VBM and lesion studies that report involvement of the DLPFC in emotion regulation ability using a self-report emotion regulation ability assessment [18], [22], [45]. Moreover, numerous fMRI studies have consistently shown increased neural activity in the DLPFC when participants are instructed to deploy a variety of emotion regulation strategies, including cognitive reappraisal and expressive suppression [13], [16], [46]. The DLPFC is known for its critical role in cognitive control, working memory, and response selection [47], [48]. Taken together, recruitment of the DLPFC may facilitate emotion regulation through a range of effortful cognitive processes, such as selecting, shifting, and maintaining regulation strategies. For example, the DLPFC may be used to direct attention to reappraisal-relevant stimulus features and hold in mind reappraisal goals as well as the content of one's reappraisal [49]. Our finding that the DLPFC has a male-specific correlation with emotion regulation ability seems to support behavioral observations that report males use more rational and detachment coping strategies in a stressful environment than females [50], [51].

In contrast, we found that females' emotion regulation ability was associated with rGMV in the anatomical cluster that extends from the left brainstem to the left hippocampus, the left amygdala and the insular cortex, which are implicated in emotional processes. Numerous fMRI studies have demonstrated that the hippocampus, the amygdala and the insular cortex are involved in successful emotion regulation [12], [13], [14], [16], [17]. In particular, the amygdala plays a critical role in detecting, as well as attending to and encoding affectively arousing and threatening stimuli into memory [52], [53], [54], and its hyperactivation is associated with a variety of affective disorders [55]. The hippocampus is involved in establishing declarative or episodic memory for autobiographic events [56]. Therefore, recruitment of the hippocampus and amygdala may help females to accurately perceive emotional events and detect cues that signal potential threats, thus facilitating their regulation of emotion. The insular cortex is a fundamental multimodal sensory integration region, and has highly reciprocal connections with cortical (e.g., the anterior cingulate and prefrontal cortex) and subcortical regions (e.g., amygdala and basal ganglia) [57]. It is involved in the experience of bodily self-awareness, emotional processing, sexual memory, and regulating autonomic functions [58], [59], [60], [61], and possibly plays a pathophysiological function in anxiety disorders and emotion dysregulation [62], [63]. The insular cortex, as well as the hippocampus and the left amygdala are central components of the limbic system that has been extensively implicated in many emotional processes such as emotional memory and emotion recognition [41], [64]. This may be consistent with the findings of previous behavioral studies that report females use emotion-focused coping strategies more frequently when under stress [50], [51].

These results may contribute to our understanding of sex differences in disorders and maladaptive behaviors that are associated with emotion regulation ability. For example, it has been reported that hippocampal and amygdala volume are smaller in depressed females than that in normal participants [65], [66], [67], [68], [69] and, more specifically, than in depressed males [70], [71], [72]. This is in accordance with our findings that rGMV in the hippocampus and the amygdala is more strongly associated with emotion regulation ability in females than in males, which in part supports the finding of a higher prevalence of affective disorders (e.g., depression) in females [73]. On the other hand, maladaptive behaviors (e.g., aggressive behavior and alcohol-related problems) are more prevalent in males [74], [75]. The finding that rGMV in the DLPFC is more strongly associated with emotion regulation ability in males than in females, similarly suggests that maladaptive behaviors might result from difficulties in the cognitive control of emotions.

In conclusion, our study provides the first evidence for the sex-specific neuroanatomical basis of emotion regulation ability. Specifically, we found that females' emotion regulation ability is associated with rGMV in emotion-focused brain regions, whereas males' emotion regulation ability tends to be associated with rGMV in brain regions implicated in cognitive processes. However, several limitations need to be addressed for future research. First, we assessed each participant's emotion regulation ability using a self-report questionnaire. Although this questionnaire predicts real-world outcomes, such as well-being and depressive symptomatology [34], it would perhaps be useful to employ objective measures of emotion regulation ability when examining sex-specific neuroanatomy involved in the process in order to examine convergence across diverse measures. Second, only healthy participants were recruited, and therefore, it is unknown whether our results relate to clinical samples. Given the relationship between emotion dysregulation and affective disorders, studying emotion regulation ability in a clinical population is necessary to improve our understanding of emotion regulation deficits and to provide insight into treatment implications. Third, this study is a cross-sectional design, which does not draw causal inferences. Future investigations should ideally utilize longitudinal designs to examine changes in emotion regulation ability over the course of development.
